# Lésion érosive du mamelon

**DOI:** 10.11604/pamj.2017.27.64.11030

**Published:** 2017-05-29

**Authors:** Amina Kissou, Badr Edine Hassam

**Affiliations:** 1 Service de Dermatologie, CHU Ibn Sina, Rabat, Maroc

**Keywords:** Paget, mamelon, érosion, Paget’s disease, male, nipple

## Image en médecine

La maladie de Paget du sein a été décrite la première fois par Sir James Paget en 1874. Il s'agit d'une pathologie qui, bien que rare, est souvent associée à un carcinome in situ ou invasif sous-jacent. Elle représente 1 à 3 % des tumeurs mammaires et moins de 5 % des cancers du sein chez l'homme. Nous rapportons le cas d'un patient âgé de 50 ans qui a consulté pour une lésion érosive du mamelon gauche évoluant depuis plus d'un an. L'examen clinique trouvait une lésion érosive de 1,5cm de diamètre qui siégeait au niveau de l'aréole du sein gauche, suintante, surmontée de croûtes en périphérie, avec disparition du relief du mamelon (A). Il n'y avait pas d'adénopathies ni masse palpable. Plusieurs diagnostics ont été évoqués: un eczéma chronique, une maladie de Paget ou encore une adénomatose érosive du mamelon. Une biopsie cutanée a été faite et avait objectivait une maladie de Paget associée à un carcinome canalaire infiltrant. Les seins étaient classées BIRADS 1 en écho-mammographie. La tomodensitométrie thoraco-abdomino-pelvienne et la scintigraphie osseuse étaient normales. Le patient a bénéficié d'une mastectomie avec curage ganglionnaire. L'histologie a objectivé une maladie de Paget avec un carcinome canalaire infiltrant et les ganglions étaient indemnes. Il a bénéficié d'une chimiothérapie et d'une radiothérapie avec bonne évolution et un recul de deux ans.

**Figure 1:**
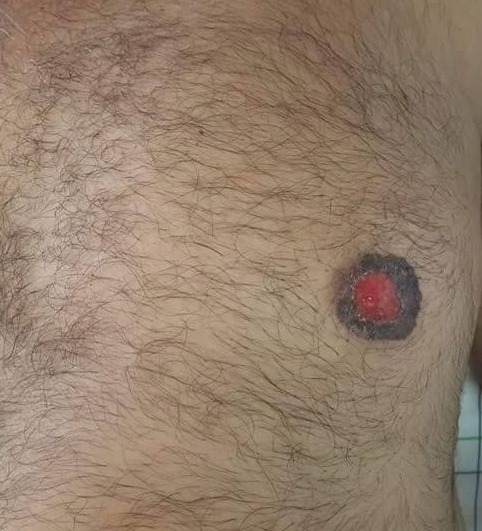
lésion érosive du mamelon avec disparition du relief de ce dernier

